# Oil-in-Water Pickering Emulsions Stabilized with Nanostructured Biopolymers: A Venue for Templating Bacterial Cellulose

**DOI:** 10.3390/ijms241713141

**Published:** 2023-08-24

**Authors:** Víctor Calvo, Laura Fuentes, Daniel Berdejo, José M. González-Domínguez, Wolfgang K. Maser, Ana M. Benito

**Affiliations:** 1 Instituto de Carboquímica (ICB-CSIC), C/Miguel Luesma Castán 4, 50018 Zaragoza, Spain; vcalvo@icb.csic.es (V.C.); fuentesvarelaura@gmail.com (L.F.); wmaser@icb.csic.es (W.K.M.); 2 Departamento de Producción Animal y Ciencia de los Alimentos, Facultad de Veterinaria, Instituto Agroalimentario de Aragón-IA2, Universidad de Zaragoza-CITA, 50013 Zaragoza, Spain; berdejo@unizar.es

**Keywords:** biopolymers, bionanofabrication, cellulose nanocrystals, chitin nanocrystals, nanocellulose

## Abstract

Pickering emulsions (PEs) differ from conventional emulsions in the use of solid colloidal particles as stabilizing agents instead of traditional amphiphilic molecules. Nanostructured biopolymers (NBs) emerge as a promising alternative for PE stabilization owing to their remarkable biocompatibility, abundant availability, and low cost. To explore this potential, a study is herein presented, in which cellulose nanocrystals (CNCs), both type I and type II allomorphs, and chitin nanocrystals (ChNCs) were used for stabilizing oil-in-water PEs prepared by the use of ultrasound. Sunflower oil was selected as the oil phase as it offers the advantages of being edible, renewable, and inexpensive. By utilizing ζ-potential, static light diffraction, and visual observations, we determined the optimal oil/water ratio for each type of NB to obtain stable emulsions after 14 days. The optimized PEs were used to form bacterial nanocellulose composites through emulsion templating. To our knowledge, this study represents a pioneering work in exploiting oil-in-water PEs for this approach. Additionally, it entails the first utilization of nonmercerized type II CNCs as stabilizers for PEs, while also establishing a direct comparison among the most relevant NBs. The resulting composites exhibited a unique morphology, composed of larger pores compared to standard bacterial nanocellulose aerogels. These findings highlight the notable potential of NBs as stabilizers for PEs and their ability to generate green nanocomposites with tailored properties.

## 1. Introduction

A classical emulsion is a mixture of two immiscible liquids stabilized with an amphiphilic molecule, known as the emulsifying agent or surfactant. An emulsion is composed of two liquids: a dispersed/internal phase and a continuous/external phase. In contrast to classical emulsions, Pickering emulsions (PEs) employ solid colloidal particles as the stabilizer [1,2], such as silica particles [1], bacterial cellulose nanofibrils [3], or cellulose nanocrystals (CNCs) [4]. PEs have been found to display better stability against coalescence and sedimentation compared to classical emulsions [1]. Different parameters, such as the surface chemistry of these particles, their concentration, or the ratio between the phases, can be modified to tune the characteristics of the PEs [1]. These have been proven as a classical emulsions substitute in diverse applications: 3D printing [5], pastry processing [6], or the creation of conductive foams [7]. 

Nanostructured biopolymers (NBs) are sustainable nanomaterials with increasing interest due to their excellent physical and chemical properties and biocompatibility together with high abundance and a low cost. Cellulose nanomaterials are the most investigated kind of NBs, being classified into three groups depending on the morphology and their dimensions: CNCs and cellulose nanofibers, which are both synthesized via top-down methodologies, and bacterial nanocellulose (BNC), which is produced in a bottom-up way by the action of specific bacteria [8,9]. These materials, due to their intrinsic anisotropy, have great potential for sustainable applications [10]. Cellulose has two main crystalline allomorphs depending on the chain orientation: type I (parallel), dominant in nature, and type II (antiparallel), which is mainly manufactured [8]. The allomorph type has a major role in the properties and applications of CNCs, for instance, unique electrochemical or biological performance when combined with carbon nanotubes [11,12,13]. Acid hydrolysis is the most convenient way to obtain both CNC main allomorphs via a one-pot synthesis, as the outcome can be controlled by changing the synthesis conditions (time and temperature of reaction, homogenization method, and timing of acid addition) [12,14].

Chitin nanocrystals (ChNCs) are synthesized, similar to CNCs, by breaking the noncrystalline parts of chitin chains via chemical or physical processes, such as TEMPO oxidation, ball milling, or acid hydrolysis [15,16]. ChNCs have a higher water stability, biocompatibility, and biodegradability compared to bulk chitin [17]. The size and shape of ChNCs is similar to type I CNCs, but ChNCs possess positive surface charges due to the protonation of some amino groups [18,19,20], while CNCs obtained by acid hydrolysis have negative charges due to the insertion of sulfate ester groups [21,22].

In recent years, NBs have been commonly used for the stabilization of PEs. In 2011, Kalashnikova et al. [23] reported the first successful use of CNCs, derived from BNC, for hexadecane in water PEs. Similarly, Tzoumaki et al. [19] demonstrated the formation of corn oil in water PEs using ChNCs. These initial studies provided a strong foundation for further exploration of NBs as effective and sustainable emulsifiers. Li et al. [24] performed the first comparison of different CNCs allomorphs for PEs. They observed that type I CNCs exhibited superior emulsifying properties compared to type II CNCs, as per a larger emulsion ratio and smaller droplet size. Notably, in their study, type II CNCs were prepared via mercerization, a traditional conversion process that removes the existing ester sulfate groups [25]. Peng et al. [18] compared type I CNCs and ChNCs and found a higher relative emulsifying capacity in the ChNCs (with similar sizes), whereas CNCs displayed better stability. However, there is still a need to investigate the use of type II CNCs prepared via acid hydrolysis (i.e., with their full content on negatively charged sulfate ester groups) for PEs formulations, as no previous studies have explored the use of this specific kind of type II CNC. Type II CNCs obtained by acid hydrolysis possess a higher number of sulfate groups in their surface compared to type I CNCs [26], which could entail potential performance differences. Additionally, a direct comparison between type II CNCs and ChNCs has not yet been addressed. Filling these two gaps would provide valuable insights leading to novel potential applications of PEs.

In particular, amongst the different NBs, BNC is a promising material in various fields due to its outstanding properties, including high tensile strength, high crystallinity, and high water-holding capacity. As mentioned earlier, BNC is a NB biosynthesized by aerobic bacteria, directly at the nanoscale in the form of hydrogel pellicles, from a carbon source, such as glucose, fructose, or glycerol [27,28,29]. However, BNC properties can be further improved by the introduction of additives into the culture media, such as vegetable oil [30]. The production of composites, which can be classified as in situ or ex situ [31], could also promote new functionalities in the BNC, for example, the incorporation of CNTs [32] or aloe vera extracts [33].

Recently, innovative methodologies have been developed for the production of BNC composites to create functional and advanced BNC-based materials [34,35,36,37]. Some strategies are the use of alginate beads to form BNC microspheres [34] and 3D printing techniques [35]. Emulsion templating is another promising method, which involves the generation of BNC capsules through water-in-oil emulsions [36]. Despite being widely known in microbiology and its high potential for enhanced BNC composites, only the aforementioned precedent work of Pepicelli et al. [36] successfully used water-in-oil emulsions for templating of BNC materials. Because the culture medium was the internal phase of the emulsions, they obtained BNC microcapsules. However, the reverse scenario (oil-in-water emulsions), with the culture medium as the external phase, remains unexplored and represents a research opportunity. Moreover, the addition of vegetable oil to the BNC culture medium reportedly increases the yield and provides improved BNC hydrogels [30,38,39]. In particular, a tailored porosity and microstructure of BNC hydrogel could be therefore envisioned, as the production of BNC would occur in the aqueous continuous phase, together with an increased yield.

**Figure 1:**
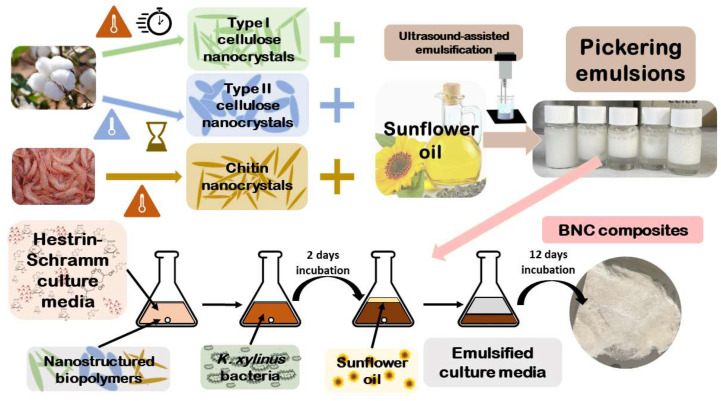
Scheme of the overall conceptual approach for preparing PEs stabilized by different biopolymer nanocrystals, and their use as BNC growth template.

Our approach aims at searching for a new paradigm for culturing BNC in a nonconventional manner, through oil-in-water PEs as a tool for sustainable bionanofabrication. Henceforth, this study (Figure 1) is devoted to employing three different NBs as stabilizers for oil-in-water PEs, with vegetable oil as the dispersed phase, in order to attain an edible and inexpensive system. Ultimately, by using an in situ strategy with the PE as a template ruling the eventual microstructure, we aim to demonstrate the potential of the optimally prepared PEs as a means for producing bottom-up “all-biopolymer” BNC nanocomposites.

## 2. Results and Discussion

### 2.1. Synthesis and Characterization of CNCs and ChNCs

**Figure 2:**
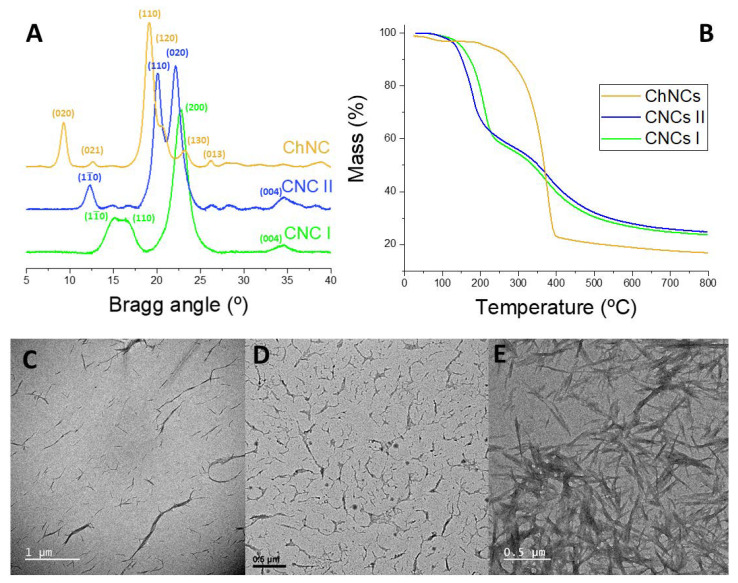
Characterization of the NBs: XRD (**A**) TGA (**B**); and TEM: CNCs I, scale bar = 1 μm (**C**), CNCs II, scale bar = 0.5 μm (**D**), and ChNCs, scale bar = 0.5 μm (**E**).

The structural, thermal, and morphological analysis of the synthesized NBs (chemical structure in Figure S1, Supplementary Materials) were performed by X-ray diffraction (XRD), thermogravimetric analysis (TGA), and transmission electron microscopy (TEM), as depicted in Figure 2. Figure 2A shows the XRD patterns of CNCs I, CNCs II, and ChNCs. The diffractogram of CNCs I exhibits a main peak corresponding to the (200) diffraction plane, along with two slightly overlapped diffraction peaks, ($1\bar{1}0$) and (110), at around 16°. CNCs II exhibit two peaks of similar intensity, (110) and (020), and two less intense peaks, ($1\bar{1}0$) and (004). Notably, both allomorphs display a peak corresponding to the (004) plane at the same Bragg angle. As for ChNCs, a distinct intense peak at 19° is observed, representing the (110) plane, with a small shoulder on its right, denoting the (120) plane. Additionally, ChNCs display several less intense peaks, corresponding to the (020), (021), (130), and (013) planes. Thus, the XRD pattern of each nanocrystal aligns with the expected phases for type I and type II CNCs and α-chitin [12,15,20,21]. The TGA analysis (Figure 2B) revealed distinct thermal degradation profiles among the NBs. CNCs I and CNCs II exhibited a similar two-step thermal degradation process, yielding a comparable mass residue of around 25%. In contrast, ChNCs showed higher thermal stability with a one-step degradation process, resulting in a lower mass residue at 800 °C compared to both CNCs. Moving to the morphological aspects, TEM microscopy (Figure 2C–E) confirmed the distinct characteristics for each type of NB. Type I CNCs fibrils were longer, type II CNCs were more twisted and shorter, and ChNCs fibers shared a similar needle shape to that of type I CNCs. These results provide insight into the uniqueness of each nanocrystal variant and contribute to a comprehensive understanding of their behavior as emulsifiers in PEs.

**Figure 3:**
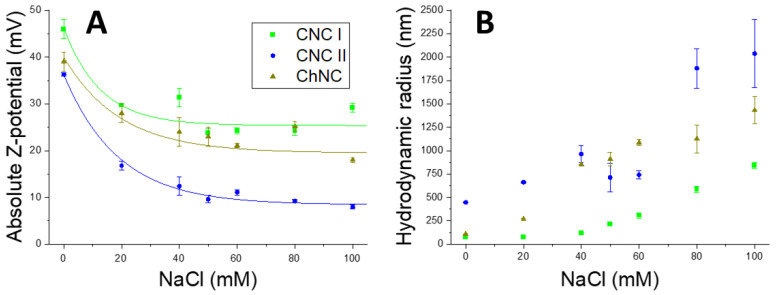
Absolute ζ-potential (**A**) and DLS (**B**) results for NBs with different NaCl concentrations. Green squares = CNCs I, blue circles = CNCs II, brown triangles = ChNCs.

The ζ-potential, which relates to the electrostatic repulsion, helps to evaluate the stability of PEs through time [2]. A high absolute ζ-potential value would cause the oil droplets to collapse as the nanocrystals would repel each other too much, while a very low value would facilitate the nanocrystals agglomeration. Therefore, to obtain a stable PE, it is necessary to slightly reduce the ζ-potential of nanocrystal dispersions below 30 mV by using an ionic compound, such as NaCl, to screen the surface charge while maintaining a suitable particle size. To determine the optimal NaCl concentration, the ζ-potential and the hydrodynamic radius of the nanocrystals were measured at different NaCl concentrations (Figure 3). Although CNCs showed a negative ζ-potential due to sulfate ester groups, and ChNCs displayed a positive ζ-potential due to amino group protonation; it is noteworthy that their absolute values were compared, as this provides valuable insights into the electrostatic repulsion irrespective of the sign. In the absence of NaCl, CNCs I exhibited the highest ζ-potential (−46 ± 2 mV), while type II CNCs and ChNCs showed similar values below 40 mV. In terms of hydrodynamic radius, type I CNCs and ChNCs displayed closely comparable values, whereas type II CNCs corresponded to larger hydrodynamic spheres, around 500 nm in size. The ζ-potential of all three NBs decreased logarithmically with increasing NaCl concentration up to a constant value. However, as the NaCl content increased, their hydrodynamic radii gradually increased after a small plateau. Since the nanocrystals agglomerated at higher NaCl concentrations, their hydrodynamic radii could not be measured beyond 100 mM NaCl. Polydispersity indexes (Figure S2, Supplementary Materials) show homogeneity at NaCl concentrations below 50 nm and a steep rise with higher ones, similarly to the hydrodynamic radius results (Figure 3). Based on our results, but also in agreement with available literature data, we identified 50 mM NaCl as the optimal concentration for our PEs formulation, because it provides adequate ζ-potential values on NBs without significantly increasing their hydrodynamic radii.

### 2.2. PEs Preparation, Optimization and Characterization

**Figure 4:**
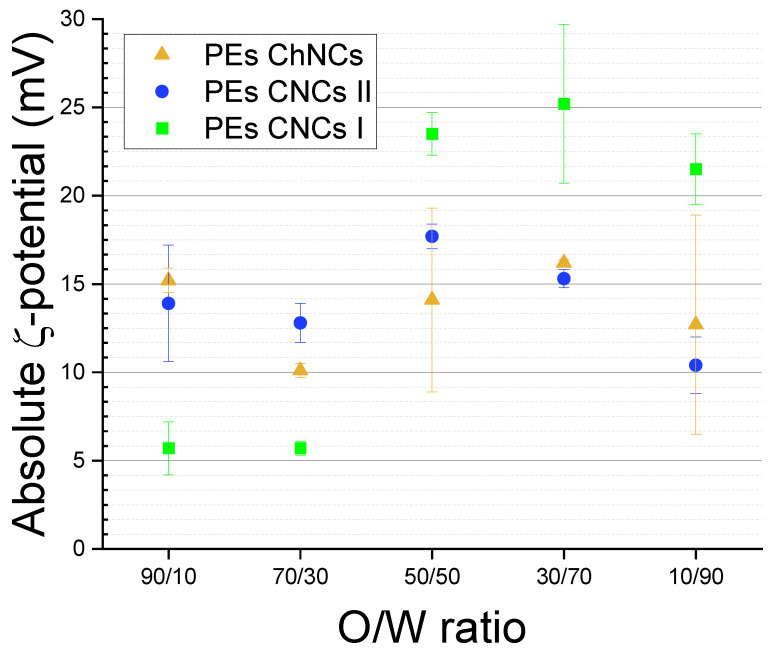
ζ-potential results for oil-in-water PEs with different oil/water ratios after preparation with different nanocrystalline NBs: CNCs I (green squares), CNCs II (blue circles), and ChNCs (yellow triangles).

Oil-in-water PEs were prepared using the three types of NB nanocrystals, type I CNCs, type II CNCs, and ChNCs, as stabilizers. Different oil/water (O/W) ratios (10/90, 30/70, 50/50, 30/70, and 90/10) were employed while maintaining a constant nanocrystal concentration of 10 g/L and 50 mM of NaCl in the aqueous phase. The objective was to identify a stable emulsion suitable for the preparation of BNC composites via an emulsion templating approach. Figure 4 displays the ζ-potential results for each O/W ratio and NB. We note that the represented data correspond to absolute values to make a clearer comparison between negatively charged CNCs and positively charged ChNCs. The ζ-potential values of the PEs obtained with O/W ratios of 90/10 and 70/30 were lower (below 15 mV in absolute numbers) than that of the bare nanocrystal aqueous colloids. This could be attributed to an excess of oil, which reduces the total NB exposure in each droplet. Interestingly, CNCs II exhibited the maximum ζ-potential value (in absolute numbers) at the O/W ratio of 50/50, while the other two NBs reached their maximum ζ-potential at the 30/70 ratio. Conversely, decreasing the O/W ratio to 10/90, the ζ-potential (in absolute value) decreased, likely due to a decrease in the number of nanocrystals exposed in the droplet surface due to the low amount of oil.

**Figure 5:**
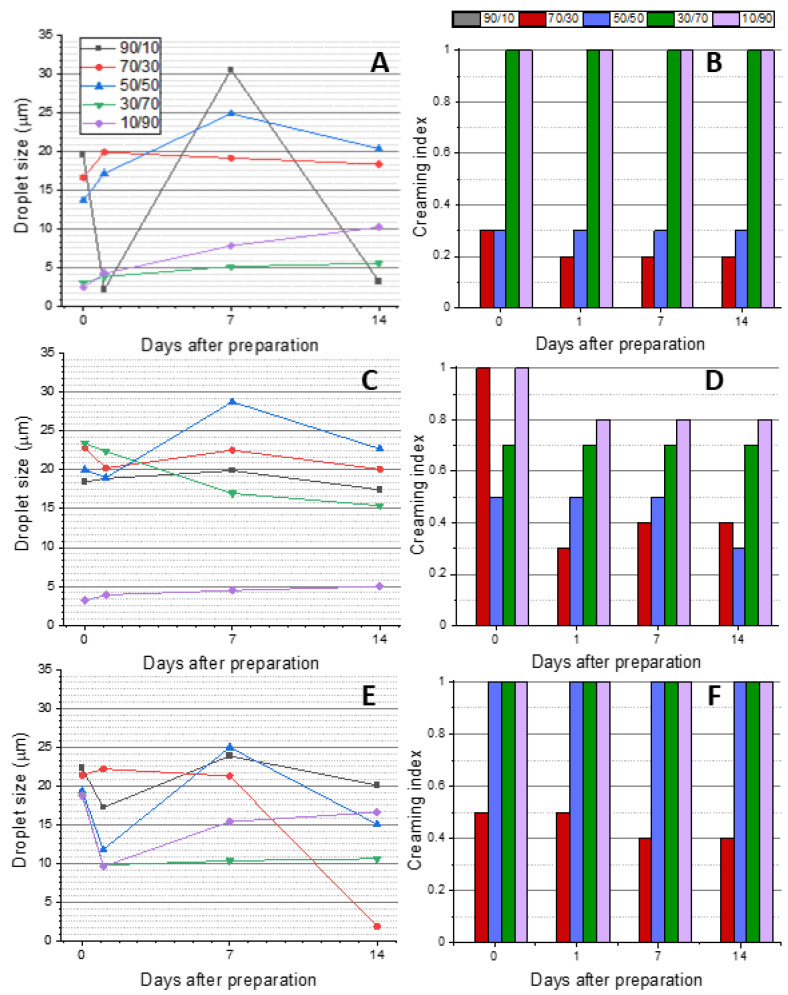
Evolution of droplet size by SLS (**A**,**C**,**E**) and creaming index (**B**,**D**,**F**) for oil-in-water PEs with different O/W ratios over time: CNCs I (**A**,**B**), CNCs II (**C**,**D**), and ChNCs (**E**,**F**). There are no CI data for any PEs with the 90/10 O/W ratio because these samples did not emulsify at all.

The droplet size evolution of the PEs was determined using static light scattering (SLS), as presented in Figure 5A,C,E. The creaming index for each PE was determined by visual observation (see Figures S3–S5, Supplementary Materials); the results are summarized in Figure 5B,D,F. Notably, the 90/10 O/W ratio resulted in unstable PEs and the SLS results exhibited significant variability, while it was not possible to measure the CI as no emulsification could be observed (see Figures S3–S5, Supplementary Materials). Similarly, the 70/30 O/W ratio also failed to yield stable emulsions due to limited NB availability, which led to a decrease in the creaming index over time. This indicated insufficient nanocrystals to effectively cover the oil/water interface, resulting in instability. Remarkably, type I CNCs required a minimum O/W ratio of 30/70 to achieve a creaming index of 1 (Figure 5B). The droplet size was significantly smaller at this ratio and at 10/90, compared to the other three ratios (Figure 5A). For type II CNCs, even at the highest O/W ratio, a creaming index of 1 could not be achieved after 14 days (Figure 5D). However, the creaming index stabilized at 0.7 for the 30/70 O/W ratio and at 0.8 for the 10/90 O/W ratio. Similarly, type II CNCs showed a substantial reduction in droplet size at the 10/90 O/W ratio (Figure 5C). In contrast, ChNCs provided the only PE with a stable creaming index of 1 when using the 50/50 O/W ratio (Figure 5F), but the SLS results (Figure 5E) demonstrated higher variability and greater stability at higher O/W ratios.

**Figure 6:**
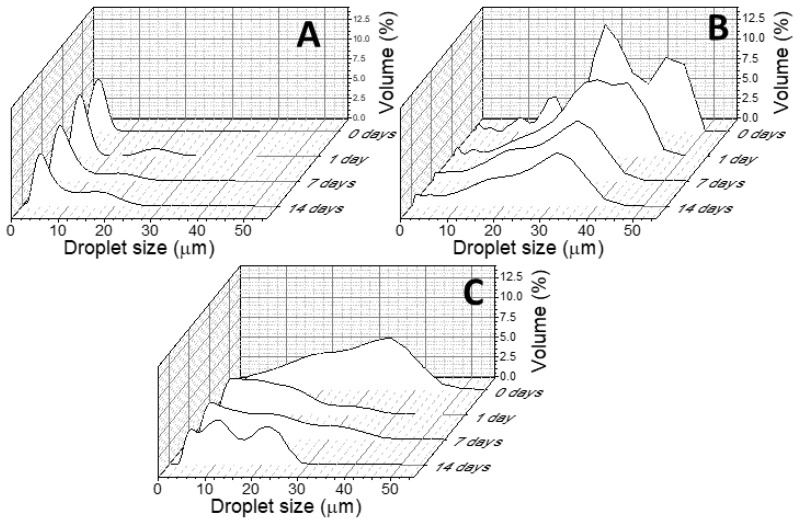
Evolution of the droplet size over time for oil-in-water PEs with 30O/70W ratio and stabilized with CNCs I (**A**), CNCs II (**B**), and ChNCs (**C**).

Based on the ζ-potential results (Figure 4) and the stability characteristics (Figure 5), the 30/70 O/W ratio emerged as the optimal choice for all three NB nanocrystals. Figure 6 presents the droplet size evolution over time at the chosen O/W ratio, where type I CNCs (Figure 6A) exhibited the smallest droplet size, below 10 μm, while type II CNCs (Figure 6B) displayed a larger mean droplet size of around 20 μm. The behavior of the PEs stabilized by ChNCs differed slightly, as the size of each droplet was decreasing over time, with final sizes falling between those of type I and type II CNCs. These results highlight the influence of the nanocrystal nature on the PEs characteristics. Type I CNCs demonstrated the finest dispersion, followed by ChNCs, while type II CNCs exhibited larger droplets. The selected 30/70 O/W ratio ensured stable emulsions for all nanocrystals in a practical time frame, and it is a commonly used O/W ratio for PEs with NBs [23]. Since this ratio has been coincident with that noted elsewhere in the literature, we postulate that the size of the nanocrystals may play a leading role compared to the surface chemistry or charge, since the three NBs herein employed are more different in terms of surface chemistry and more similar in terms of size, and yet they have provided the same optimal O/W ratio in this specific oil-in-water system.

### 2.3. BNC Production using PEs as Template

**Figure 7:**
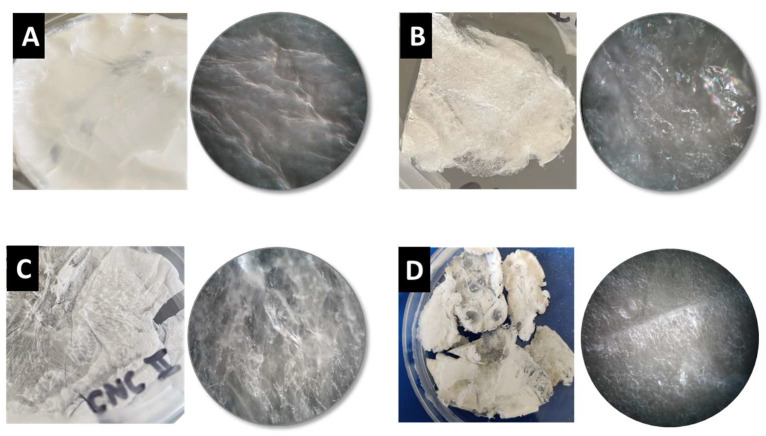
Photographs (squares) and optical microscopy images (circles, obtained at 5× and numerical aperture of 0.13) of BNC composites with different culture media: regular HS (**A**), emulsified culture media with CNCs I (**B**), with CNCs II (**C**), and with ChNCs (**D**).

**Figure 8:**
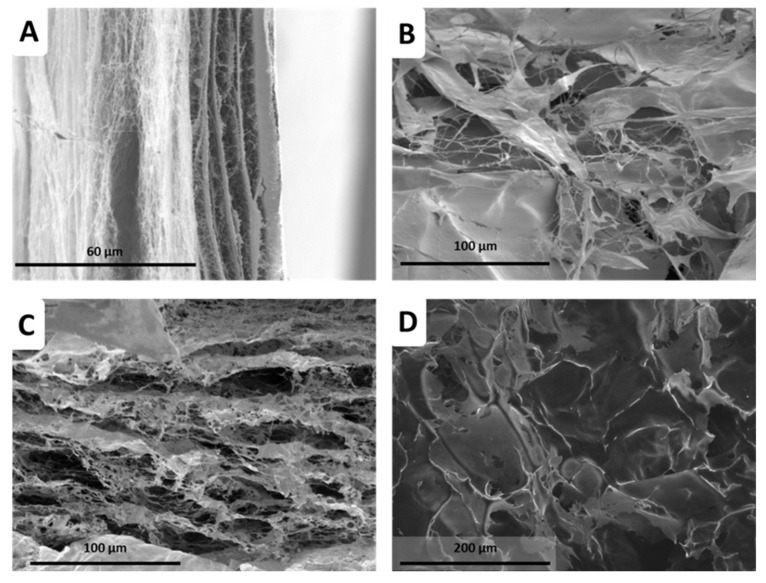
SEM images of BNC composites with different culture media: regular HS (**A**), emulsified culture media with CNCs I (**B**), with CNCs II (**C**), and with ChNCs (**D**).

In the pursuit of BNC production using PEs as templates, we prepared a regular Hestrin–Schramm (HS) culture medium and three different emulsified culture media (one for each NB) and seeded them with *K. xylinus* bacteria. After 12 days, BNC hydrogels were extracted, cleaned, and freeze-dried to obtain aerogels; a graphical summary of the approach can be found in the experimental section. Surprisingly, a visual inspection of the BNC aerogels (Figure 7) revealed significant textural differences compared to those typically obtained with conventional HS culture media, both at the macroscopic and microscopic scales. Notably, these BNC composites exhibited higher porosity than conventional BNC (Figure 7). Similarly, scanning electron microscopy (SEM) images (Figure 8) further support the striking porous microstructure obtained via emulsion templating, highlighting the distinctive characteristics achieved through this innovative approach when compared to previous works [29,30,31,33,40].

BNC aerogels produced under normal conditions (Figure 8A) presented laminar structure with entangled cellulose nanofibrils forming small pores of less than 1 µm [29]. As aerogels are obtained after the emulsion templating strategy, the porous microstructure drastically changes, ending in much larger pores, with a less entangled aspect and the presence of small foils. This suggests the aerogels to be BNC composites, as similar morphologies have been observed with ex situ strategies [41]. For both CNC cases (Figure 8B,C), the microstructure size distribution is nearly coincident to that of the PE droplet sizes during emulsion templating (Figure 6), the larger pores being 15–20 µm diameter for CNC-I, and about 40–50 µm for CNC-II. The case of ChNCs seems to display a microstructure half way in between those of CNC-I and CNC-II. This suggests an actual templating effect of PEs during the BNC growth process.

**Figure 9:**
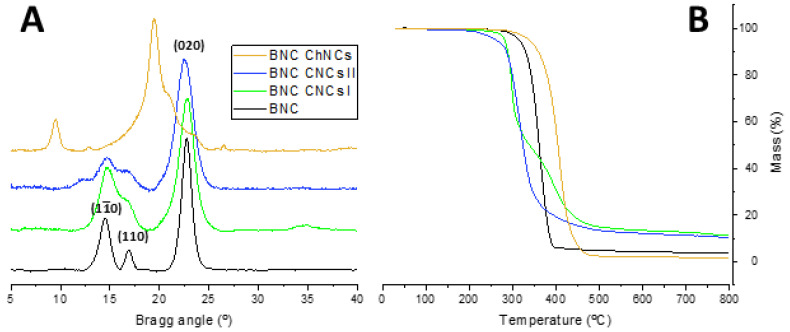
Characterization by XRD (**A**) and TGA (**B**) of BNC composites prepared by emulsion templating with different PE-based culture media.

The BNC aerogels were further characterized using XRD and TGA analysis (Figure 9). The XRD analysis (Figure 9A) showed the presence of the main diffraction planes associated with BNC in the samples prepared with CNCs I and II, which are characteristic of type I cellulose and commonly observed in previous works of BNC composites [33,34,42]. In contrast, the XRD pattern of the BNC-ChNCs nearly coincided with that of pure ChNCs, and the characteristic peaks of BNC seemed to be absent, suggesting that ChNCs remain remarkably well integrated, even after the hydrogel cleaning process, heavily wrapping the BNC scaffold. This composite must have BNC beneath due to the macroscopic aspect, but ChNCs hide the BNC features because of their higher crystallinity and amount. In the case of the BNC-CNCs II sample, a slight shift to lower Bragg angles of the main peak corresponding to plane (200) was observed due to the presence of the plane (020) from type II CNCs, confirming their successful incorporation into the BNC composites (Figure 9A). Another intrinsic feature from CNC-II observed in the XRD diffractogram, is the small peak at ~12°, providing further evidence of the achievement of a BNC nanocomposite, in this case with CNC-II as the filler.

The TGA results (Figure 9B) of BNC composites with both types of CNCs showed lower temperature of maximum degradation rate and higher residue percentage compared to a regular BNC aerogel (bare HS-mediated). Interestingly, the BNC-CNCs I composite displayed a two-step degradation process, while the BNC-CNCs II composite exhibited single-step degradation with a lower onset temperature (Figure 9B). In contrast, the incorporation of ChNCs significantly increased the thermal stability of the BNC-ChNCs composite compared to regular BNC (Figure 9B). Consistent with the XRD patterns, the degradation of BNC-ChNCs presented an analogous profile to that of ChNCs alone (Figure 2B), but with a significant rise of nearly 50 °C in the maximum degradation rate temperature. This striking outcome highlights the great potential of these specific BNC composites in applications where improved thermal properties are desired.

Overall, the physical characterization of BNC aerogel composites underscores the remarkable potential of PE-based templating to tailor the native BNC microstructure by controlling droplet size and long-term stability of emulsified culture media. The resulting materials after the bacterial nanofabrication process exhibited distinct uniqueness based on each NB employed for the emulsions: while both CNCs provide a BNC composite in which BNC is the predominant component (and nanocrystals appear as fillers), ChNCs, in contrast, end in aerogels in which they emerge as the major component, exhibiting structural and thermal behavior similar to pure ChNCs, but integrated in a BNC-based structure. To support this premise, it is important to remark that direct addition of ChNCs to the bacterial culture medium with no PE formation results in progressive sedimentation of ChNCs (Figure S6, Supplementary Materials), quickly hindering their contact with the air–liquid interface and hence drastically reducing their eventual incorporation into the BNC. Therefore, the PE-based approach opens exciting possibilities to precisely tailor the structure and properties of BNC materials to meet specific application needs. For instance, new or improved uses of BNC could arise in membrane technology (e.g., gas or ion permeation, liquid filtration) or in environmental remediation (e.g., water purification, pollutant retention), as long as the specific porosity requirements can be satisfied by this PE strategy. Such versatility establishes the way for developing custom-designed BNC composites.

## 3. Materials and Methods

### 3.1. Materials and Reagents

Microcrystalline cellulose powder, 20 μm (#310697), chitin extracted from shrimp shells (practical grade, powder; C7170), concentrated H_2_SO_4_ (98%), glucose, peptone, yeast extract, citric acid, CaCO_3_, and NaH_2_PO_4_ were purchased from Merck (Barcelona, Spain). Hydrochloric acid 37% (AGR IS; CHAC-0AI) was purchased from Labkem (Barcelona, Spain). NaCl for analysis (CAS: 7647-14-5) was purchased from Scharlab (Barcelona, Spain). Sunflower oil was purchased from a local supermarket (Zaragoza, Spain). *Komagataeibacter xylinus* strain (NCIMB 5346) was purchased from CECT (Valencia, Spain) in fresh state (CECT 473). Ultrapure water, obtained from a Siemens Ultraclear device (Cole Palmer, Cambridgeshire, UK), was used for all the experiments.

### 3.2. Synthesis of NBs

The synthesis of CNCs from microcrystalline cellulose powder was performed following an in-house protocol based on acid hydrolysis described in a recent work [14]. By varying the synthesis conditions, it was possible to select the allomorph outcome (type I or type II) while maximizing the yield and minimizing the overall time. Type I CNCs were synthesized using a fast addition (shorter than 5 min) of H_2_SO_4_ followed by a 10 min reaction at 70 °C. On the other hand, type II CNCs were produced with a slow H_2_SO_4_ addition and a 1 h reaction at ambient temperature. After the reaction, each type of CNC dispersion was subjected to a process of neutralization and purification based on decanting, dialysis, and centrifugation.

The synthesis of ChNCs is based on typical acid hydrolysis of chitin with 3M hydrochloric acid [15]. Briefly, 4 g of chitin powder were added to 80 mL 3M HCl and the mixture was kept under reflux at 100 °C. Afterward, the solution was poured into 1 L of ultrapure water and decantated overnight in a refrigerator at 4 °C. ChNCs were neutralized by dialysis against ultrapure water and the liquid was centrifuged at 9000 rpm (9327 rcf) for 20 min, keeping the liquid and twice redispersing the solid with ultrapure water.

CNC and ChNC dispersions were characterized by dynamic light scattering (DLS) and ζ-potential diluting using NaCl solutions between 0 and 100 mM to study the effect of the salt in the NBs dispersions (hydrodynamic radii and ζ-potential). The dispersions were observed via TEM, lyophilized to determine the yield and concentration, and the solids were further analyzed by XRD and TGA.

### 3.3. Characterization of NBs

DLS and ζ-potential were carried out in a nanosizer device (Malvern Nano ZS instrument, IESMat, Madrid, Spain), using the respective refractive index of each biopolymer. TEM images were taken on a JEOL JEM-2100F (Tokio, Japan) model EM-20014 with a 200 kV field-emission gun (Schottky) and an ultrahigh resolution pole piece (UHR). NBs dispersions were deposited over a carbon-coated Ni grid (200 mesh), purchased from Aname (La Rioja, Spain). XRD patterns of the xerogels were measured in Bragg–Brentano geometry in the range 2θ = [5–40°] with a Bruker D8 Advance diffractometer (Boston, MA, USA) using a Cu tube as the X-ray source with 40 kV tube voltage and 40 mA current. TGA was measured in a Netzsch TG 209F1 device (Selb, Germany), in a nitrogen atmosphere, starting at ambient temperature and rising to 800 °C (heating ramp 10 °C/min).

### 3.4. Preparation of Oil-in-Water PEs

Different amounts of NBs dispersions, ultrapure water, NaCl, and sunflower oil were mixed to a final volume of 10 mL. The NB concentration was 10 g/L in the aqueous phase for all PEs, and the optimal NaCl concentration was determined to be 50 mM (vide infra). Afterward, an ultrasound tip (Hielscher DRH-P400S; 400 W maximum acoustic power; 24 kHz maximum frequency at 60% amplitude, and 50% cycle time) was used to induce the emulsification for 4 min; the tip was located at the top part of the mixture. Different oil/water ratios (90/10, 70/30, 50/50, 30/70, and 10/90) were studied to observe the effect of this parameter in the final properties.

### 3.5. Characterization of Oil-in-Water PEs

The ζ-potential of each PE was measured after preparation in the same device that was used for the NB dispersions, but diluting in a 50 mM NaCl solution. The droplet size of the oil-in-water PEs was determined by SLS in a Beckman Coulter LS 13 320 with a laser, diluting in a 50 mM NaCl solution. PEs were stored in test tubes for two weeks and measured by SLS the same day as prepared, the following day, one week after, and two weeks after. Photographs were also taken to calculate the creaming index [43] and to compare the visual appearance. Creaming index was determined by the ratio between the height of the emulsion and the total length of the mixture, and to evaluate the storage stability of the PEs.

### 3.6. Production of BNC Composites

The optimally stable conditions for PEs for each NB were used to prepare an emulsified culture medium for the production of BNC. A regular liquid culture medium was prepared with Hestrin–Schramm (HS) culture, which is the most widely used for BNC production, as a reference or blank [44]. This culture medium comprises glucose (2%), yeast extract (0.5%), peptone (0.5%), citric acid (0.26%), and NaH_2_PO_4_ (0.115%).

**Figure 10:**
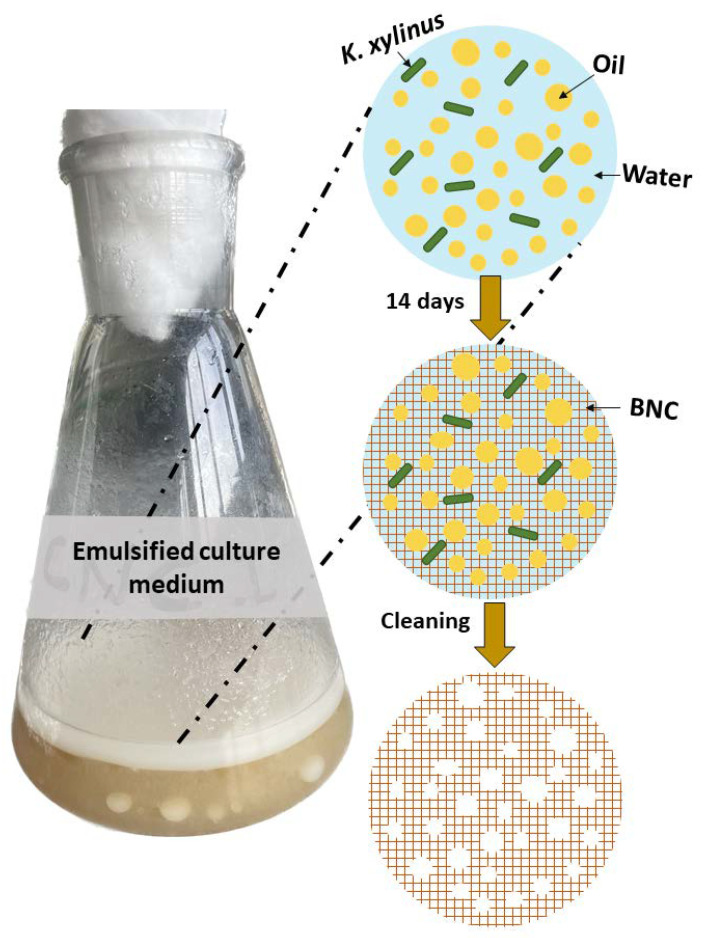
Summary of the emulsion templating approach for the synthesis of BNC-NBs composites.

Each emulsified culture medium was prepared by dissolving the HS components and 50 mmol of NaCl in a 10 g/L NB solution. Then, these aqueous phases were sterilized and inoculated with *K. xylinus* bacteria. After two days of incubation at 30 °C to increase the bacteria population, 30 mL of sunflower oil was added and the PEs were formed using a sterilized ultrasound tip for 20 min (Hielscher DRH-P400S; 400 W maximum power; 24 kHz maximum frequency at 60% amplitude, and 50% cycle time). After two weeks at 30 °C, the BNC hydrogels produced by bacteria were extracted from the 4 culture media and subjected to a clean-up process (Figure 10). The first step was washing with ultrapure water at 100 °C for 40 min. This was followed by 4 repeated washing cycles with NaOH 0.1 M for 20 min and neutralization with ultrapure water, periodically changing the water until reaching neutral pH [40] (Figure S7). The clean hydrogels were lyophilized to obtain aerogels. These were subjected to different characterization techniques: XRD, TGA, and SEM.

### 3.7. Characterization of BNC Composites

BNC composites were analyzed by XRD and TGA. Optical microcopy images were acquired in a ZEISS Axio Imager A1, through an objective of 5× and a numerical aperture of 0.13. SEM equipment, a JSM 6400 device working at 20 kV and with a maximum point-to-point resolution of 3.5 nm, was used to visualize the surface of BNC composites.

## 4. Conclusions

The outcome of this study demonstrates the successful utilization of NBs as stabilizers for oil-in-water PEs, providing new insights into the properties of the three investigated NBs. It is also a promising avenue for the preparation of novel BNC composites via emulsion templating. Our findings indicate that the selected oil/water ratio of 30/70 provided stable emulsions for all three NB nanocrystals, able to host the bionanofabrication activity of *K. xylinus* bacteria, eventually resulting in BNC aerogels with distinct textural properties compared to those obtained through unmodified HS culture media. This makes the aerogels potentially useful in a wide range of applications (e.g., environmental remediation, tailored separation, etc.). The XRD analysis unequivocally confirms the incorporation of type II CNCs and ChNCs into the BNC composites. Moreover, the TGA highlights the extraordinarily enhanced thermal stability of the BNC-ChNC composites when compared to regular BNC aerogels. In conclusion, this study illuminates the use of NBs as PEs stabilizers and initializes the synthesis of BNC composites through oil-in-water emulsion templating. The successful integration of the NBs and the unique morphologies attained on these “all nanobiopolymer” composites will encourage their future study as tailored membranes in the field of clean energy and environmental sciences.

## Data Availability

The data will be available upon request.

## Supplementary Materials

The supporting information can be downloaded at: https://www.mdpi.com/article/10.3390/ijms241713141/s1.

## References

[1] de Carvalho-Guimarães F.B., Correa K.L., de Souza T.P., Rodríguez Amado J.R., Ribeiro-Costa R.M., Silva-Júnior J.O.C. (2022). A Review of Pickering Emulsions: Perspectives and Applications. Pharmaceuticals.

[2] Low L.E., Siva S.P., Ho Y.K., Chan E.S., Tey B.T. (2020). Recent Advances of Characterization Techniques for the Formation, Physical Properties and Stability of Pickering Emulsion. Adv. Colloid Interface Sci..

[3] Zhang X., Wang D., Liu S., Tang J. (2022). Bacterial Cellulose Nanofibril-Based Pickering Emulsions: Recent Trends and Applications in the Food Industry. Foods.

[4] Dai H., Wu J., Zhang H., Chen Y., Ma L., Huang H., Huang Y., Zhang Y. (2020). Recent Advances on Cellulose Nanocrystals for Pickering Emulsions: Development and Challenge. Trends Food Sci. Technol..

[5] Ma T., Cui R., Lu S., Hu X., Xu B., Song Y., Hu X. (2022). High Internal Phase Pickering Emulsions Stabilized by Cellulose Nanocrystals for 3D Printing. Food Hydrocoll..

[6] Xie Y., Lei Y., Rong J., Zhang X., Li J., Chen Y., Liang H., Li Y., Li B., Fang Z. (2021). Physico-Chemical Properties of Reduced-Fat Biscuits Prepared Using O/W Cellulose-Based Pickering Emulsion. LWT.

[7] Mougel J.B., Bertoncini P., Cathala B., Chauvet O., Capron I. (2019). Macroporous Hybrid Pickering Foams Based on Carbon Nanotubes and Cellulose Nanocrystals. J. Colloid Interface Sci..

[8] Klemm D., Cranston E.D., Fischer D., Gama M., Kedzior S.A., Kralisch D., Kramer F., Kondo T., Lindström T., Nietzsche S. (2018). Nanocellulose as a Natural Source for Groundbreaking Applications in Materials Science: Today’s State. Mater. Today.

[9] Kargarzadeh H., Mariano M., Gopakumar D., Ahmad I., Thomas S., Dufresne A., Huang J., Lin N. (2018). Advances in Cellulose Nanomaterials. Cellulose.

[10] Benselfelt T., Kummer N., Nordenström M., Fall A.B., Nyström G., Wågberg L. (2023). The Colloidal Properties of Nanocellulose. ChemSusChem.

[11] Dortez S., Sierra T., Álvarez-Sánchez M.Á., González-Domínguez J.M., Benito A.M., Maser W.K., Crevillen A.G., Escarpa A. (2022). Effect of Nanocellulose Polymorphism on Electrochemical Analytical Performance in Hybrid Nanocomposites with Non-Oxidized Single-Walled Carbon Nanotubes. Microchim. Acta.

[12] González-Domínguez J.M., Ansón-Casaos A., Grasa L., Abenia L., Salvador A., Colom E., Mesonero J.E., García-Bordejé J.E., Benito A.M., Maser W.K. (2019). Unique Properties and Behavior of Nonmercerized Type-II Cellulose Nanocrystals as Carbon Nanotube Biocompatible Dispersants. Biomacromolecules.

[13] González-Domínguez J.M., Grasa L., Frontiñán-Rubio J., Abás E., Domínguez-Alfaro A., Mesonero J.E., Criado A., Ansón-Casaos A. (2022). Intrinsic and Selective Activity of Functionalized Carbon Nanotube/Nanocellulose Platforms against Colon Cancer Cells. Colloids Surf. B Biointerfaces.

[14] Calvo V., Álvarez Sánchez M.Á., Güemes L., Martínez-Barón C., Baúlde S., Criado A., González-Domínguez J.M., Maser W.K., Benito A.M., Miguel A.A. (2023). Preparation of Cellulose Nanocrystals: Controlling the Crystalline Type by One-Pot Acid Hydrolysis. ACS Macro Lett..

[15] Zhong T., Wolcotta M.P., Liua H., Wang J. (2019). Developing chitin nanocrystals for flexible packaging coatings. Carbohydr. Polym..

[16] Yang T., Qi H., Liu P., Zhang K. (2020). Selective Isolation Methods for Cellulose and Chitin Nanocrystals. ChemPlusChem.

[17] Bai L., Liu L., Esquivel M., Tardy B.L., Huan S., Niu X., Liu S., Yang G., Fan Y., Rojas O.J. (2022). Nanochitin: Chemistry, Structure, Assembly, and Applications. Chem. Rev..

[18] Peng G., Wu D. (2022). Insight into Different Roles of Chitin Nanocrystals and Cellulose Nanocrystals towards Stabilizing Pickering Emulsions. Food Hydrocoll..

[19] Tzoumaki M.V., Moschakis T., Kiosseoglou V., Biliaderis C.G. (2011). Oil-in-Water Emulsions Stabilized by Chitin Nanocrystal Particles. Food Hydrocoll..

[20] Li J., Revol J.F., Marchessault R.H. (1997). Effect of Degree of Deacetylation of Chitin on the Properties of Chitin Crystallites. J. Appl. Polym. Sci..

[21] Xing L., Gu J., Zhang W., Tu D., Hu C. (2018). Cellulose I and II Nanocrystals Produced by Sulfuric Acid Hydrolysis of Tetra Pak Cellulose, I. Carbohydr. Polym..

[22] Han J., Zhou C., Wu Y., Liu F., Wu Q. (2013). Self-Assembling Behavior of Cellulose Nanoparticles during Freeze-Drying: Effect of Suspension Concentration, Particle Size, Crystal Structure, and Surface Charge. Biomacromolecules.

[23] Kalashnikova I., Bizot H., Cathala B., Capron I. (2011). New Pickering Emulsions Stabilized by Bacterial Cellulose Nanocrystals. Langmuir.

[24] Li X., Li J., Gong J., Kuang Y., Mo L., Song T. (2018). Cellulose Nanocrystals (CNCs) with Different Crystalline Allomorph for Oil in Water Pickering Emulsions. Carbohydr. Polym..

[25] Lin N., Dufresne A. (2014). Surface Chemistry, Morphological Analysis and Properties of Cellulose Nanocrystals with Gradiented Sulfation Degrees. Nanoscale.

[26] Flauzino Neto W.P., Putaux J.L., Mariano M., Ogawa Y., Otaguro H., Pasquini D., Dufresne A. (2016). Comprehensive Morphological and Structural Investigation of Cellulose I and II Nanocrystals Prepared by Sulphuric Acid Hydrolysis. RSC Adv..

[27] Calvo V., González-Domínguez J.M., Benito A.M., Maser W.K. (2022). Synthesis and Processing of Nanomaterials Mediated by Living Organisms. Angew. Chem. Int. Ed..

[28] Esa F., Tasirin S.M., Rahman N.A. (2014). Overview of Bacterial Cellulose Production and Application. Agric. Agric. Sci. Procedia.

[29] Calvo V., Torrubia J., Blanco D., García-Bordeje E., Maser W.K., Benito A.M., González-Domínguez J.M. (2020). Optimizing Bacterial Cellulose Production towards Materials for Water Remediation. NATO Science for Peace and Security Series B: Physics and Biophysics.

[30] Żywickaa A., Junka A.F., Szymczykc P., Chodaczekd G., Grzesiake J., Sedghizadehf P.P., Fijałkowski K. (2018). Bacterial cellulose yield increased over 500% by supplementation of medium with vegetable oil. Carbohydr. Polym..

[31] Stumpf T.R., Yang X., Zhang J., Cao X. (2018). In Situ and Ex Situ Modifications of Bacterial Cellulose for Applications in Tissue Engineering. Mater. Sci. Eng. C.

[32] Park S., Park J., Jo I., Cho S.P., Sung D., Ryu S., Park M., Min K.A., Kim J., Hong S. (2015). In Situ Hybridization of Carbon Nanotubes with Bacterial Cellulose for Three-Dimensional Hybrid Bioscaffolds. Biomaterials.

[33] Piaia L., Pittella C.Q.P., De Souza S.S., Berti F.V., Porto L.M. (2022). Incorporation of Aloe Vera Extract in Bacterial Nanocellulose Membranes. Polimeros.

[34] Kim J.H., Park S., Kim H., Kim H.J., Yang Y.-H., Kim Y.H., Jung S.-K., Kan E., Lee S.H. (2017). Alginate/Bacterial Cellulose Nanocomposite Beads Prepared Using *Gluconacetobacter xylinus* and Their Application in Lipase Immobilization. Carbohydr. Polym..

[35] Schaffner M., Rühs P.A., Coulter F., Kilcher S., Studart A.R. (2017). 3D Printing of Bacteria into Functional Complex Materials. Sci. Adv..

[36] Pepicelli M., Binelli M.R., Studart A.R., Rühs P.A., Fischer P. (2021). Self-Grown Bacterial Cellulose Capsules Made through Emulsion Templating. ACS Biomater. Sci. Eng..

[37] Lu H., Sun S., Sun J., Peng X., Li N., Ullah M.W., Zhang Y., Chen L., Zhou J. (2023). Sustainable production of flocculant-containing bacterial cellulose composite for removal of PET nano-plastics. Chem. Eng. J..

[38] Żywickaa A., Wenelska K., Junka A., Czajkowska J., Fijałkowski K. (2019). An efficient method of Yarrowia lipolytica immobilization using oil-and emulsion-modified bacterial cellulose carriers. Electron. J. Biotechnol..

[39] Pongjinapeth T., Sudying P., Jaturapiree P. (2020). The utilization of wastewater of Thai fermented rice noodle (Kanom-jeen) manufacturing process for the production of bacterial cellulose by Acetobacter xylinim TISTR 975. IOP Conf. Ser. Mater. Sci. Eng..

[40] Zeng M., Laromaine A., Roig A. (2014). Bacterial Cellulose Films: Influence of Bacterial Strain and Drying Route on Film Properties. Cellulose.

[41] Kanno T., Uyama H. (2018). Unique Ivy-like Morphology Composed of Poly(Lactic Acid) and Bacterial Cellulose Cryogel. ACS Omega.

[42] Stoica-Guzun A., Stroescu M., Jinga S.I., Mihalache N., Botez A., Matei C., Berger D., Damian C.M., Ionita V. (2016). Box-Behnken experimental design for chromium(VI) ions removal by bacterial cellulose-magnetite composites. Int. J. Biol. Macromol..

[43] McClements D.J. (2007). Critical Review of Techniques and Methodologies for Characterization of Emulsion Stability. Crit. Rev. Food Sci. Nutr..

[44] Hestrin S., Schramm M. (1954). Synthesis of Cellulose by *Acetobacter xylinum*. 2. Preparation of Freeze-Dried Cells Capable of Polymerizing Glucose to Cellulose. Biochem. J..

