# Supplementary material for: Oil-in-Water Pickering Emulsions Stabilized with Nanostructured Biopolymers: A Venue for Templating Bacterial Cellulose
Source: Int J Mol Sci. 2023 Aug 24;24(17):13141. doi: 10.3390/ijms241713141 (PMC10487472; doi:10.3390/ijms241713141)
Supplement: Supplementary file 1 [file ijms-24-13141-s001.zip › ijms-2554718-supplementary.pdf]

Article

Oil-in-Water Pickering Emulsions Stabilized with Nanostructured Biopolymers: a Venue for Templating Bacterial Cellulose

Víctor Calvo [1], Laura Fuentes [1], Daniel Berdejo [2], José M. González-Domínguez [1,*], Wolfgang K. Maser [1] and Ana M. Benito [1,*]

1 Instituto de Carboquímica (ICB-CSIC), C/ Miguel Luesma Castán 4, 50018 Zaragoza, Spain; vcalvo@icb.csic.es (V.C.); fuentesvarelaura@gmail.com (L.F.); jmgonzalez@icb.csic.es (J.M.G.D.); wmaser@icb.csic.es (W.K.M.); abenito@gmail.com (A.M.B.)

2 Departamento de Producción Animal y Ciencia de los Alimentos, Facultad de Veterinaria, Instituto Agroalimentario de Aragón-IA2 (Universidad de Zaragoza-CITA), Zaragoza, 50013, Spain; berdejo@unizar.es

* Correspondence: jmgonzalez@icb.csic.es (J.M.G.D.); abenito@icb.csic.es (A.M.B.)

Chemical structure of the nanostructured biopolymers

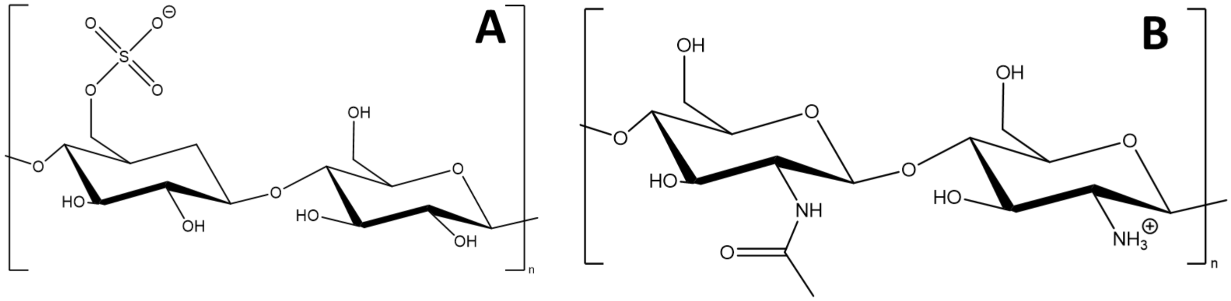

Figure S1. Chemical structure of cellulose nanocrystals (A) and chitin nanocrystals (B).

Polydispersity results of nanostructured biopolymers

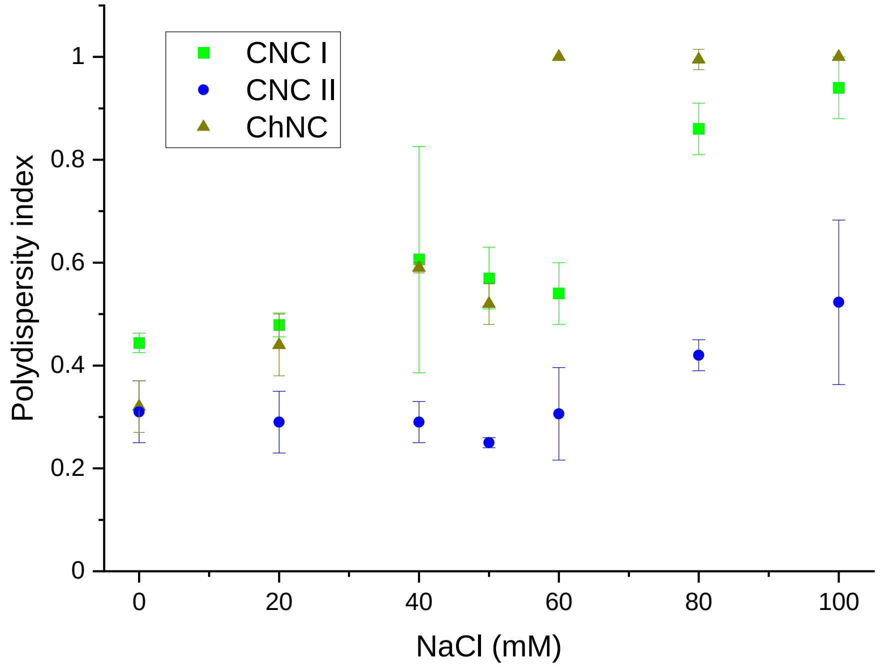

Figure S2. Polydispersity indexes of the NBs (CNCs I, CNCs II and ChNCs) in water with different NaCl concentrations.

Evolution of the visual appearance of PEs

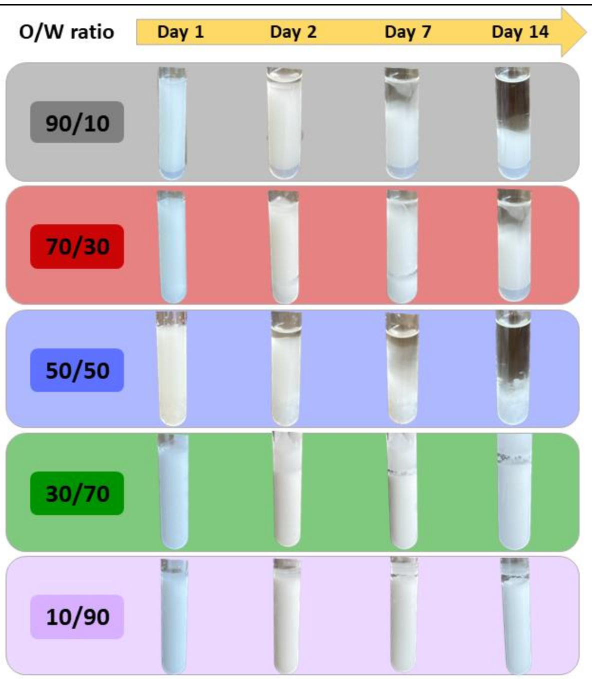

Figure S3. Evolution over time of the PEs made with CNCs I. The images were used to determine the creaming index.

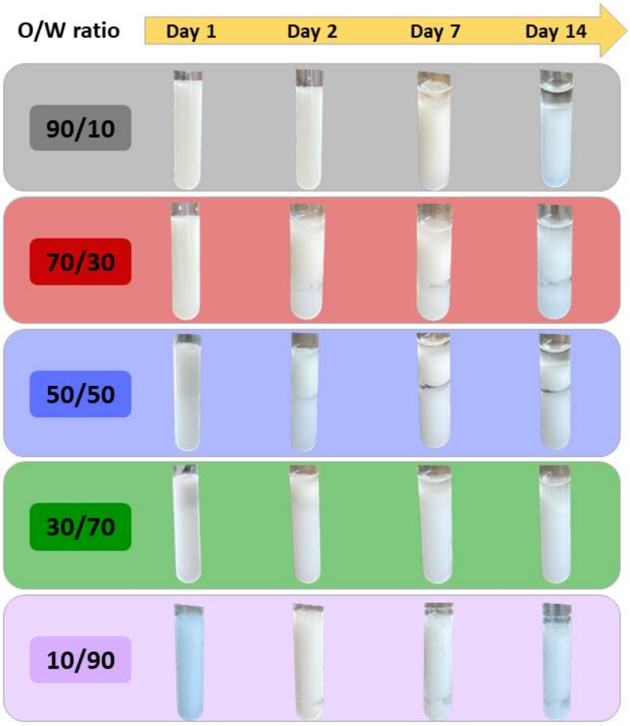

Figure S4. Evolution over time of the PEs made with CNCs II. The images were used to determine the creaming index.

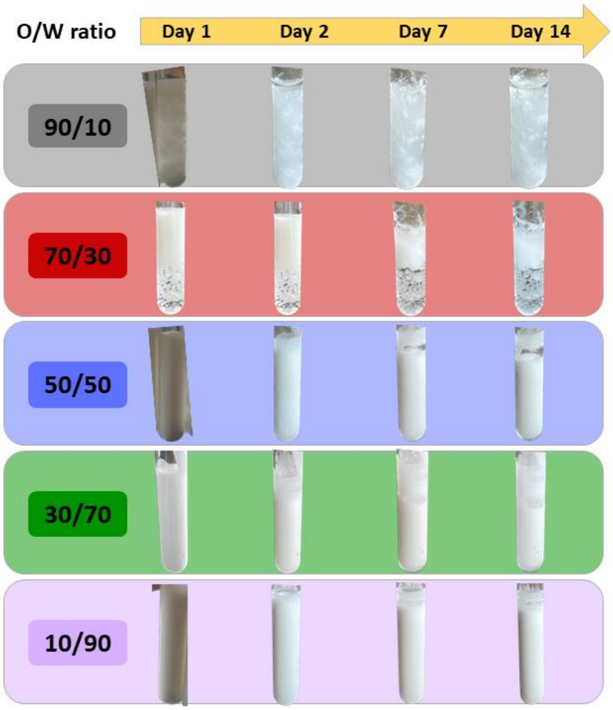

Figure S5. Evolution over time of the PEs made with ChNCs. The images were used to determine the creaming index.

BNC production with ChNCs without PEs

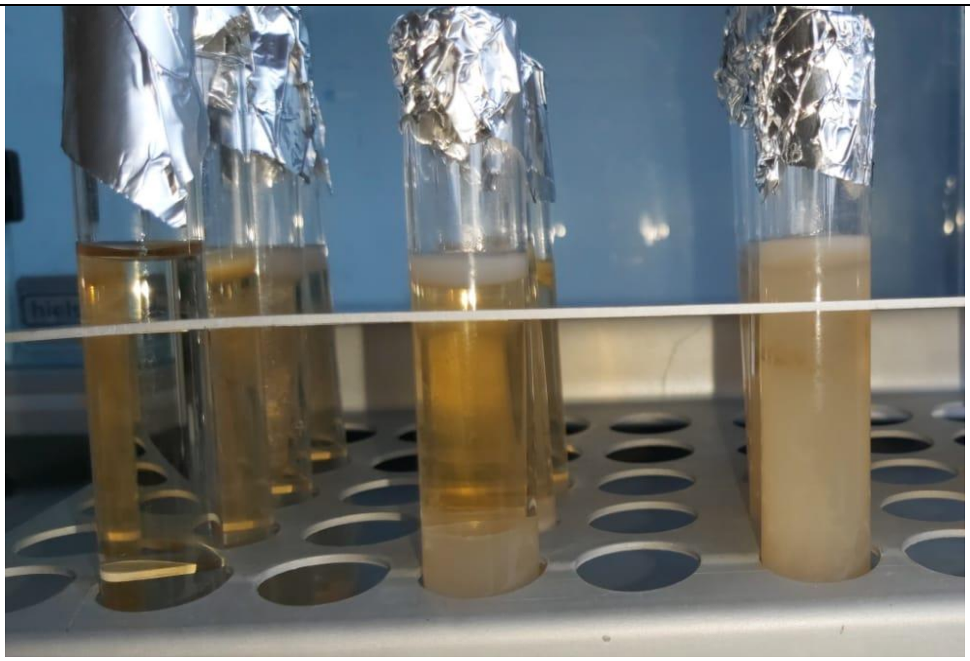

Figure S6. BNC culture media without ChNCs (left) and two different ChNCs concentrations (center and right) after 14 days since inoculation. It can be observed that ChNCs precipitate, what reduces the ChNCs incorporation in the BNC composites.

BNC hydrogels prepared with emulsified culture media

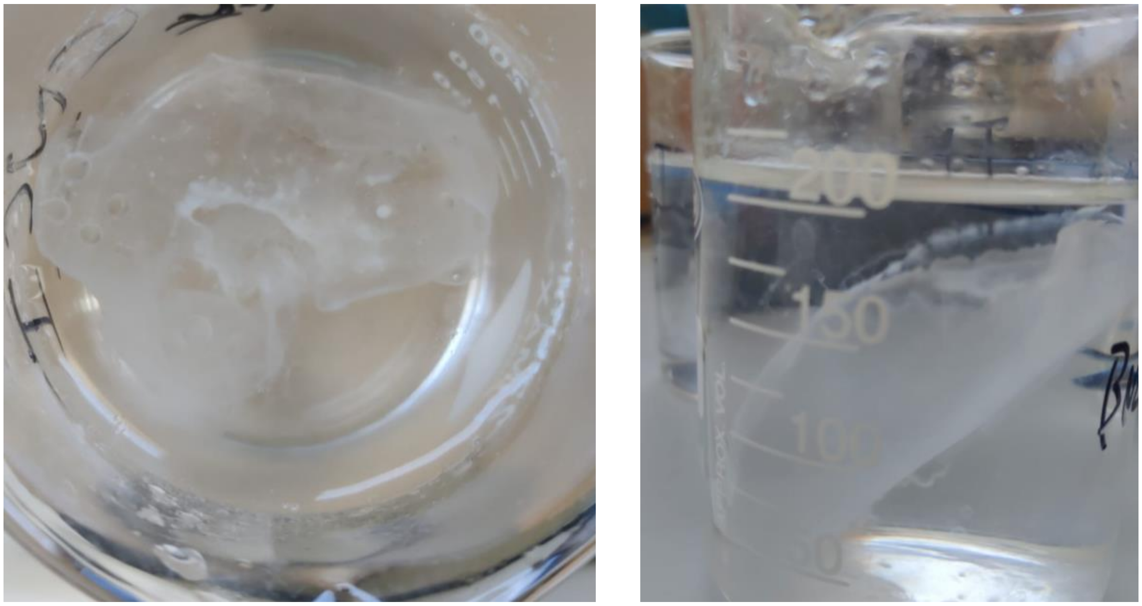

Figure S7. BNC hydrogels prepared with emulsified culture media after cleaning.
